# Supplementary material for: The Polish adaptation of the work role performance questionnaire
Source: PLoS One. 2025 Oct 6;20(10):e0332609. doi: 10.1371/journal.pone.0332609 (PMC12500121; doi:10.1371/journal.pone.0332609)
Supplement: Attachment — Submitted filename: response_letter.docx [file pone.0332609.s001.docx]

**Reviewer #1 Response**:

We sincerely thank the Reviewer for their thorough and constructive evaluation of our manuscript. We greatly appreciate the recognition of the study’s contribution and methodological strengths. The Reviewer’s insightful comments have been invaluable in identifying areas for conceptual clarification, methodological refinement, and clearer reporting. We believe that addressing these points has strengthened the clarity and overall quality of the revised manuscript.

_____________________________________________________________________________

**Comment #1**: 1. Conceptual and Theoretical Issues
Mismatch Between Hypotheses and Findings:
The expected relationships between predictors (e.g., self-efficacy with proficiency roles, openness with adaptivity) were largely unsupported. The authors acknowledge these inconsistencies but do not sufficiently engage with their theoretical implications. A deeper reconsideration of the cultural interpretation of adaptivity and proficiency is warranted, rather than attributing discrepancies solely to "contextual shifts."

**Response**: We thank the Reviewer for this insightful comment. In the revised manuscript, we expanded our discussion of the unexpected findings to address their theoretical implications more thoroughly. Specifically, in the subsection “Predictors of Work Role Performance”, we provide a deeper cultural interpretation of the observed discrepancies. For self-efficacy, we highlight how, in uncertainty-avoidant contexts such as Poland, self-efficacy may align more strongly with adaptive rather than proficiency roles. For openness to change, we explain that in the Polish context this trait may manifest through intellectual curiosity and refinement of established procedures, which aligns more closely with proficiency than adaptivity. Finally, regarding work organization, we discuss how structured environments may foster proactive behaviors within existing frameworks rather than supporting individual adaptivity. These additions, we believe, provide a more grounded interpretation of the cultural differences, and we hope that they adequately address the Reviewer’s comment.

_____________________________________________________________________________

**Comment #2**: Premature Application of CFA Without EFA:
Given the cross-cultural adaptation context, exploratory factor analysis (EFA) should have preceded CFA. Direct application of CFA assumes structural invariance, which cannot be presumed when adapting psychological constructs across cultures.

**Response**: We thank the Reviewer for this important methodological comment. In line with the suggestion, we conducted an exploratory factor analysis prior to the confirmatory factor analysis to avoid presuming structural invariance. The results of the EFA, together with updated Table 1, have now been added to the Results section. The analysis confirmed sampling adequacy (KMO = .88; Bartlett’s χ²(26) = 947.05, p < .001), and all loadings exceeded the recommended threshold of .35 (Hair Jr et al., 2021). Only one item demonstrated cross-loading, but as the difference between the loadings exceeded .20 and both items belonged to the same role level, this was not deemed problematic for subsequent CFA. We hope that these additions address the Reviewer’s concern and provide stronger empirical support for the measurement structure.

_____________________________________________________________________________

**Comment #3**: 2. Statistical and Methodological Concerns
Handling of Non-Normal Data:
Although significant deviations from normality (e.g., Shapiro–Wilk test, high kurtosis for ITP items) are reported, the authors continue using maximum likelihood (ML) estimation without adjustment. Given these deviations, robust estimation methods (e.g., MLR or WLSMV) would have been more appropriate and should be employed or at least discussed.

**Response**: We sincerely thank the Reviewer for this valuable comment, which allowed us to improve the methodological rigor of our analyses. In response to this concern, we have replaced the maximum likelihood (ML) estimator with a more appropriate estimator for non-normal data throughout the manuscript.

First, in the CFA, we now use the robust weighted least squares estimator (WLSMV), as it is considered suitable for ordinal and non-normally distributed data. This change led to an adjustment in fit indices, which are now updated in Table 2. The revised text in the Results section reflects this modification accordingly.

Second, for the path analysis conducted to test Hypothesis 2, we also replaced the ML estimator with the diagonally weighted least squares (DWLS) estimator, as it is recommended for models involving non-normal continuous data (Baghdarnia et al., 2014; Mîndrilă, 2010).

We would like to note that the results of the path analysis remained unchanged after switching to the DWLS estimator. This is likely due to the relatively low skewness and kurtosis of the variables involved (Byrne, 2010; George & Mallery, 2010; Hair et al., 2010). We have added an explanation and appropriate citations in the revised manuscript to clarify this methodological choice.

References:

Baghdarnia, M., Doostian, Y., & Farrokhi, F. (2014). A simulation study of parameter estimation methods in structural equation modeling under ordinal and non-normal data. International Journal of Statistics and Probability, 3(3), 54–67.

Mîndrilă, D. (2010). Maximum likelihood (ML) and diagonally weighted least squares (DWLS) estimation procedures: A comparison of estimation bias with ordinal and multivariate non-normal data. IJDS, 5(1), 60–78.

Byrne, B. M. (2010). Structural equation modeling with Mplus: Basic concepts, applications, and programming. Routledge/Taylor & Francis Group.

George, D., & Mallery, M. (2010). SPSS for Windows Step by Step: A Simple Guide and Reference (17.0 Update, 10th Edition). Pearson.

Hair, J. F., Black, W. C., Babin, B. J., & Anderson, R. E. (2010). Multivariate Data Analysis (7th ed.). Pearson.

_____________________________________________________________________________

**Comment #4**: Model Fit Interpretation:
The SRMR index in Study 2 exceeded acceptable thresholds (>0.08), suggesting potential model misspecification. The authors’ justification for this (speculating about parsimony or low inter-factor correlations) is not empirically tested. Sensitivity analyses with alternative modeling approaches are recommended.

**Response**: We appreciate the Reviewer’s careful observation regarding the SRMR value in Study 2. In response to the previous comment on the handling of non-normal data, we revised our analytical approach and re-estimated the CFA using the WLSMV estimator. As a result of this change, the SRMR value is now within acceptable thresholds, and no longer suggests model misspecification. The revised model fit indices and residuals—including the updated SRMR—are presented in Table 2 of the manuscript. _____________________________________________________________________________

**Comment #5**: 3. Specific Technical Errors and Minor Points
Typographical Error:
Page 17 refers incorrectly to "PMA" roles instead of "OMA" (organization member adaptivity).

**Response**: We thank the Reviewer for this careful observation. The typographical error has been corrected, and the term now accurately refers to "OMA"

_____________________________________________________________________________

**Comment #6**: Hypotheses Operationalization:
Hypotheses about "stronger associations" (e.g., H2a–H2g) are vague. The manuscript should specify clear operational criteria (e.g., statistically significant higher standardized coefficients).

**Response**: We thank the Reviewer for this thoughtful and important observation. We agree that hypotheses involving the relative strength of associations should be clearly operationalized to ensure they are empirically testable.

In response, we revised the wording of Hypotheses 2a–2g to explicitly refer to statistically significant differences in the magnitude of standardized effects, providing clearer criteria for evaluating support. Correspondingly, we clarified our analytical approach in the manuscript: to test whether the associations of theoretically focal predictors (e.g., conscientiousness, role clarity, perceived team or organizational support) with the outcome variables were significantly stronger than those of other predictors, we conducted asymptotic z-tests comparing dependent standardized effects that share a common dependent variable. This method is based on Fisher’s z-transformation and the procedure proposed by Meng et al. (1992), which allows for valid comparisons between correlated effects in the same model structure.

To maintain clarity and interpretability, we applied this method systematically across hypotheses to compare the focal predictor with the most relevant alternative predictors, reflecting meaningful theoretical contrasts. All results of these comparisons—including z-values and p-values—are now reported explicitly in the results section (subsection “Differential Work Roles Predictors”). We hope this addresses the Reviewer’s concern and improves the clarity and rigor of our hypothesis testing.

Meng, X.-l., Rosenthal, R., & Rubin, D. B. (1992). Comparing correlated correlation coefficients. Psychological Bulletin, 111(1), 172–175. <https://doi.org/10.1037/0033-2909.111.1.172>

_____________________________________________________________________________

**Comment #7**: Redundancy:
There are repeated references to Griffin et al. (2007) within individual sections that could be consolidated for conciseness.

**Response:** We appreciate the Reviewer’s observation regarding potential redundancy in our references to Griffin et al. (2007). In response, we carefully reviewed the manuscript and revised the relevant sections to improve conciseness and clarity. Specifically, we ensured that all citations now follow the APA 7th edition guidelines: the year is included with each parenthetical citation, while in narrative citations, the year is omitted after the first mention within a single paragraph. This adjustment should enhance the readability of the text while maintaining proper attribution and consistency throughout the manuscript.
